# Molecular phylogenetics and mitogenomics of three avian dicrocoeliids (Digenea: Dicrocoeliidae) and comparison with mammalian dicrocoeliids

**DOI:** 10.1186/s13071-020-3940-7

**Published:** 2020-02-13

**Authors:** Suleman, Mian Sayed Khan, Vasyl V. Tkach, Nehaz Muhammad, Dong Zhang, Xing-Quan Zhu, Jun Ma

**Affiliations:** 1 State Key Laboratory of Veterinary Etiological Biology, Key Laboratory of Veterinary Parasitology of Gansu Province, Lanzhou Veterinary Research Institute, Chinese Academy of Agricultural Sciences, Lanzhou, 730046 Gansu People’s Republic of China; 2 Department of Zoology, University of Swabi, Swabi, 23340 Khyber Pakhtunkhwa Pakistan; 3 Department of Biology, University of North Dakota, Grand Forks, ND 58202-9019 USA; 4 Key Laboratory of Aquaculture Disease Control, Ministry of Agriculture, and State Key Laboratory of Freshwater Ecology and Biotechnology, Institute of Hydrobiology, Chinese Academy of Sciences, Wuhan, 430072 Hubei People’s Republic of China; 5 Jiangsu Co-innovation Center for the Prevention and Control of Important Animal Infectious Diseases and Zoonoses, Yangzhou University College of Veterinary Medicine, Yangzhou, 225009 Jiangsu People’s Republic of China

**Keywords:** Dicrocoeliidae, Nucleotide diversity, Mitochondrial genomes, Molecular phylogeny

## Abstract

**Background:**

The Dicrocoeliidae are digenetic trematodes mostly parasitic in the bile ducts and gall bladder of various avian and mammalian hosts. Until recently their systematics was based on morphological data only. Due to the high morphological uniformity across multiple dicrocoeliid taxa and insufficient knowledge of relative systematic value of traditionally used morphological characters, their taxonomy has always been unstable. Therefore, DNA sequence data provide a critical independent source of characters for phylogenetic inference and improvement of the system.

**Methods:**

We examined the phylogenetic affinities of three avian dicrocoeliids representing the genera *Brachylecithum*, *Brachydistomum* and *Lyperosomum*, using partial sequences of the nuclear large ribosomal subunit (28S) RNA gene. We also sequenced the complete or nearly complete mitogenomes of these three isolates and conducted a comparative mitogenomic analysis with the previously available mitogenomes from three mammalian dicrocoeliids (from 2 different genera) and examined the phylogenetic position of the family Dicrocoeliidae within the order Plagiorchiida based on concatenated nucleotide sequences of all mitochondrial genes (except *trnG* and *trnE*).

**Results:**

Combined nucleotide diversity, Kimura-2-parameter distance, non-synonymous/synonymous substitutions ratio and average sequence identity analyses consistently demonstrated that *cox*1, *cytb*, *nad*1 and two rRNAs were the most conserved and *atp*6, *nad*5, *nad*3 and *nad*2 were the most variable genes across dicrocoeliid mitogenomes. Phylogenetic analyses based on mtDNA sequences did not support the close relatedness of the Paragonimidae and Dicrocoeliidae and suggested non-monophyly of the Gorgoderoidea as currently recognized.

**Conclusions:**

Our results show that fast-evolving mitochondrial genes *atp*6, *nad*5 and *nad*3 would be better markers than slow-evolving genes *cox*1 and *nad*1 for species discrimination and population level studies in the Dicrocoeliidae. Furthermore, the Dicrocoeliidae being outside of the clade containing other xiphidiatan trematodes suggests a need for the re-evaluation of the taxonomic content of the Xiphidiata.

## Background

The Dicrocoeliidae Looss, 1899 is a highly diverse, cosmopolitan family of digenetic trematodes containing 47 genera and more than 400 species [1, 2]. Adult dicrocoeliids typically parasitize the bile ducts and gall-bladder of various amniotic vertebrates, but may rarely inhabit other organs [1, 3]. This group of digeneans includes parasites of livestock and humans causing dicrocoeliasis (*Dicrocoelium* spp.), eurytremiasis (*Eurytrema* spp.) and platynosomiasis (*Platynosomum* spp.) [4–7]. In her latest revision of the family, Pojmańska [3] distinguished four subfamilies based on the relative position of gonads, vitellarium and uterus. The dicrocoeliid systematics is very complicated and controversial due to their high phenotypic plasticity, morphological homogeneity across multiple genera, poor descriptions and improper fixation of specimens [1, 8–10]. Therefore, DNA sequence data from the nuclear or mitochondrial genome provide a complementary or alternative source of characters for the diagnostics and taxonomy of the Dicrocoeliidae.

Until recently, molecular phylogenetic studies included only a few representatives of the Dicrocoeliidae [11, 12], although the situation has been changing lately with a greater number of dicrocoeliid taxa being included in such analyses [1, 7, 10, 13–15]. Among other systematic and nomenclatural changes, the molecular results have demonstrated the non-monophyletic nature of the majority of dicrocoeliid subfamilies, which resulted in the abandonment of the subfamily-based structure of the family [1].

The majority of the previously published molecular phylogenetic studies were based on partial sequences of the nuclear large ribosomal subunit gene (28S rDNA) which has also been used for similar studies in a broad diversity of other digenean taxa [12, 16, 17]. Therefore, in the present study, we obtained 28S rDNA sequences from three avian dicrocoeliids belonging to three different genera (*Brachylecithum*, *Brachydistomum* and *Lyperosomum*) from Pakistan in order to establish their phylogenetic affinities within the family. Although 28S sequences proved to be useful for phylogenetic inference and in some cases provided phylogenies congruent with those resulting from the analyses based on mitochondrial (mt) genomes [18] within digenean families or even superfamilies, the use of a single, relatively conserved nuclear DNA marker may not be sufficient for analysis of interrelationships among closely related taxa [18]. Therefore, additional molecular markers with greater variability are needed to evaluate the genetic relationships at this taxonomic level.

Mitochondrial genomes have been proven to be a useful source of genetic markers for taxonomic identification, studies of inter- and intra-specific variation as well as systematics and phylogenetic analysis of trematodes at different taxonomic levels including members of the Dicrocoeliidae [19, 20]. Although molecular phylogenetic analyses based on complete mt genomes from multiple taxa within the Dicrocoeliidae are not yet available, mt genomes of three dicrocoeliids, *Dicrocoelium chinensis*, *D. dendriticum* and *Eurytrema pancreaticum* (all parasitic in mammals) have been published recently [19, 20]. In the present study, the complete mt genome of *Lyperosomum longicauda* Rudolphi, 1809 (the type-species of the genus) and nearly complete mitogenomes of *Brachydistomum* sp. and *Brachylecithum* sp. were sequenced and annotated. We used these mt genomes for comparison with other mammalian dicrocoeliids and for a broader phylogenetic analysis including other selected trematode taxa.

## Methods

### Parasite collection and genomic DNA isolation

Multiple specimens of adult dicrocoeliids were collected from the gall-bladder and bile ducts of different birds in the Swabi District, Khyber Pakhtunkhwa Province, Pakistan. Fifteen specimens of *L. longicauda* were obtained from the house crow *Corvus splendens* Vieillot and the rufous treepie *Dendrocitta vagabunda* (Latham), five specimens of *Brachydistomum* sp. from the robin accentor *Prunella rubeculoides* (Moore) and 100 specimens of *Brachylecithum* sp. from the shikra *Accipiter badius* Gmelin. Live specimens were killed with hot water and preserved in 80% ethanol [21]. The specimens for light microscopy examination were stained with alum carmine following the recommended protocol [21] and identified morphologically to the species- or genus-level (Additional file 1: Figure S1) based on identification keys and descriptions [3, 22, 23]. To confirm the taxonomic identity, genomic DNA of a single individual of each species was extracted following the protocol described by Gasser et al. [24], using the Wizard^®^ SV Genomic DNA Purification System (Promega, Madison, USA) according to the manufacturer’s instructions. For *L. longicauda*, genomic DNA was extracted from two adult parasites, one from each species of host.

### Amplification and analysis of nuclear 28S rDNA

An approximately 1300 bp long fragment at the 5′-end of the 28S gene of three avian dicrocoeliid species was amplified, as described in previous studies [1, 15] using the primers LSU5 and 1500R [12, 25] with an annealing temperature of 55 °C. For *L. longicauda*, DNA of two specimens (one from each host species) was used for the amplification of 28S gene in order to ensure that different isolates of the same species are identical. PCR products were purified with EZNA Gel Extraction Kit (OMEGA Bio-tek Inc., Doraville, GA, USA). Purified PCR products were sent to Genewiz Company (Beijing, China) for sequencing. Contiguous nucleotide sequences were assembled using DNAstar v7.1 [26] and Clustal X 1.83 [27] softwares and deposited in the GenBank database under the accession numbers MK685270, MK685272 and MK685269 for *L. longicauda*, *Brachydistomum* sp. and *Brachylecithum* sp., respectively.

To assess the phylogenetic interrelationships of our specimens within the Dicrocoeliidae, the newly generated 28S rDNA sequences were aligned with the matching sequences of 20 dicrocoeliid species available in GenBank using MEGA7 [28]. *Encyclometra colubrimurorum* was used as the outgroup based on the results of previous analyses [1, 12]. The trimmed sequences were 1175 bp long, including only a few gaps (1–3 nucleotides each). Phylogenetic analysis was conducted using the Bayesian inference (BI) method as implemented in MrBayes Ver. 3.2.6 [29]. Based on the Akaikeʼs information criterion, GTR+I+G was identified as the best-fitting model using jModelTest 2 software [30]. BI was conducted as follows: two Metropolis-coupled Markov chain Monte Carlo (MCMC) chains were run for 10,000,000 generations, the first 25% trees were treated as ‘burn-in’ and the final 75% of trees were used for calculating Bayesian posterior probabilities. The phylograms were visualized in FigTree ver. 1.4 software [31] and annotated in Adobe Illustrator^®^.

### Long-PCR-based sequencing of mt genomes

Degenerate primer pairs were designed based on relatively conserved regions of the mtDNA sequences of *D. chinensis*, *D. dendriticum* [19] and *E. pancreaticum* [20] and used to amplify the mitogenomes of three dicrocoelids (Additional file 2: Table S1). The complete mt genome of *L. longicauda* and the nearly complete mt genomes of *Brachydistomum* sp. and *Brachylecithum* sp. (excluding *trnG*, NCRs and *trnE*) were amplified in five or six overlapping fragments. Long PCR reactions were performed in reaction mixtures of 28 μl, containing 12.5 μl ddH_2_O, 12.5 μl PrimeStar Max DNA polymerase premix (Takara, Dalian, China), 1 μl (10–40 ng) of template DNA and 1 μl (25 µM) of each primer. PCR cycling conditions were as follows: 98 °C for 2 min; 10 cycles of 92 °C for 10 s; 50–57 °C for 30 s; 68 °C for 1 min/kb followed by 92 °C for 2 min; 22 cycles of 92 °C for 10 s; 50–57 °C for 30 s; 68 °C for 1 min/kb and a final extension for 10 min at 68 °C. Positive amplicons were sequenced at Genewiz sequencing company.

### mtDNA sequence assembly, annotation and analyses

After quality-proofing and BLASTn analysis, the three mtDNA genomes were manually assembled with the aid of DNAstar v7.1 program [26] and further aligned with published dicrocoeliid mitogenomes: mtDNA of *Brachydistomum* sp. and *Brachylecithum* sp. were aligned with the complete mt genome of *D. chinensis* (GenBank: KF318786) while *L. longicauda* mtDNA was aligned with that of *E. pancreaticum* (GenBank: KP241855) using MAFFT Ver 7.122 [32] to determine genome organization and approximate gene boundaries. The selection of reference mitogenomes was based on the published phylogenetic relationships among dicrocoeliids [1, 15]. Protein-coding genes (PCGs) and the two mt ribosomal RNAs (*rrnL* and *rrnS*) were identified *via* comparison with homologs using MAFFT Ver 7.122. The size and secondary structures of transfer RNAs (tRNAs) were identified using ARWEN [33] and MITOS [34] or by pairwise comparisons with previously annotated dicrocoeliid mitogenomes. The annotations of each mitogenome were saved as text documents which were further processed in PhyloSuite v1.1.14 [35] in order to generate the NCBI submission file (*.sqn), tables of comparisons and statistics for mitogenomes. The same software was used to translate the nucleotide sequences of 12 mitochondrial PCGs into amino acids and to calculate codon usage and relative synonymous codon usage (RSCU) for the 12 PCGs. The codon usage and RSCU for 12 PCGs of the six dicrocoeliid mitogenomes (three newly sequenced avian and three previously published mammalian) were computed and used to draw the RSCU figure using the ggplot2 [36] plugin of PhyloSuite.

Mutation rate (non-synonymous/synonymous, dN/dS) ratios among the 12 PCGs of the three newly sequenced dicrocoeliid mitogenomes were calculated using DnaSP v.5 [37]. DnaSP v.5 was also used to conduct sliding window analysis implementing window size of 300 bp and a step size of 30 bp, to estimate the nucleotide divergence between 12 PCGs, two rRNAs and 20 tRNAs (excluding *trnG* and *trnE*) of the six dicrocoeliid mitogenomes. The nucleotide contents of dicrocoeliid mitogenomes were compared with other families of the suborder Xiphidiata using PhyloSuite; the resulting files were further used to make the line plots of A + T content in ggplot2. Tandem Repeats Finder [38] and mreps [39] were used to find the tandem repeats (TRs) within the NCRs of complete mitogenome of *L. longicauda*. The secondary structures of TRs were predicted using Mfold software [40].

### Phylogenetic relationships of the Dicrocoeliidae with other digeneans

Phylogenetic analyses were conducted using all six dicrocoeliid mitogenomes and mitogenomes of 20 other digeneans from the suborder Opisthorchiata La Rue, 1957, Pronocephalata Olson, Cribb, Tkach, Bray & Littlewood, 2003, Xiphidiata Olson, Cribb, Tkach, Bray & Littlewood, 2003 and Echinostomata La Rue, 1926. A species belonging to the order Diplostomida, *Schistosoma japonicum*, was used as the outgroup. The nucleotide sequences of 12 PCGs, 2 rRNAs, and 20 tRNAs (*trnG* and *trnE* were excluded from analyses, as these two tRNAs were not sequenced for two out of three species) of all included mitogenomes were obtained from GenBank files using PhyloSuite [35]. Phylogenetic analyses were conducted using codon-based alignment of nucleotide sequences of 12 PCGs + Q-INS-i strategy of alignment for rRNAs and tRNAs. Nucleotide sequences of each gene were aligned in batches using MAFFT Ver 7.122, ambiguously aligned regions were deleted using Gblocks 0.91b [41]; sequences were subsequently concatenated into a single alignment used to generatehttps://eproofing.springer.com/journals_v2/index.php?token=GsTHfr8z9G_W7jV7z5wzAQrcLzIrKUUYWq59wmNCH8k nexus files in PhyloSuite. Phylogenetic analyses were performed using maximum likelihood (ML) and BI methods. Based on the Akaikeʼs information criterion, as implemented in ModelFinder [42], GTR+F+R5 and GTR+F+I+G4 were chosen as best-fitting models for nucleotide evolution for the ML and BI analysis, respectively. ML phylogenies were inferred using IQ-TREE [43] by performing ultrafast bootstraps [44] with 5000 replicates. BI phylogenies were inferred using MrBayes 3.2.6 [29] (with default settings) using two MCMC chains for 3,000,000 generations and 1000 sample frequency; the initial 25% (750) trees were discarded as ‘burn-in’. In addition, in order to remove effects of possible mutation saturation due to silent mutations we have performed a phylogenetic analysis based on alignment of translated sequences of 12 PCGs. This phylogeny was inferred using Jones+I+G+F model (2 parallel runs, 1,000,000 generations) using the same software and procedures as described above for the nucleotide-based phylogeny. Finally, phylograms were visualized and annotated by iTOL [45] and Adobe Illustrator® with the aid of dataset files generated by PhyloSuite.

## Results and discussion

### Molecular phylogeny within the Dicrocoeliidae based on 28S rDNA sequences

**Fig. 1:**
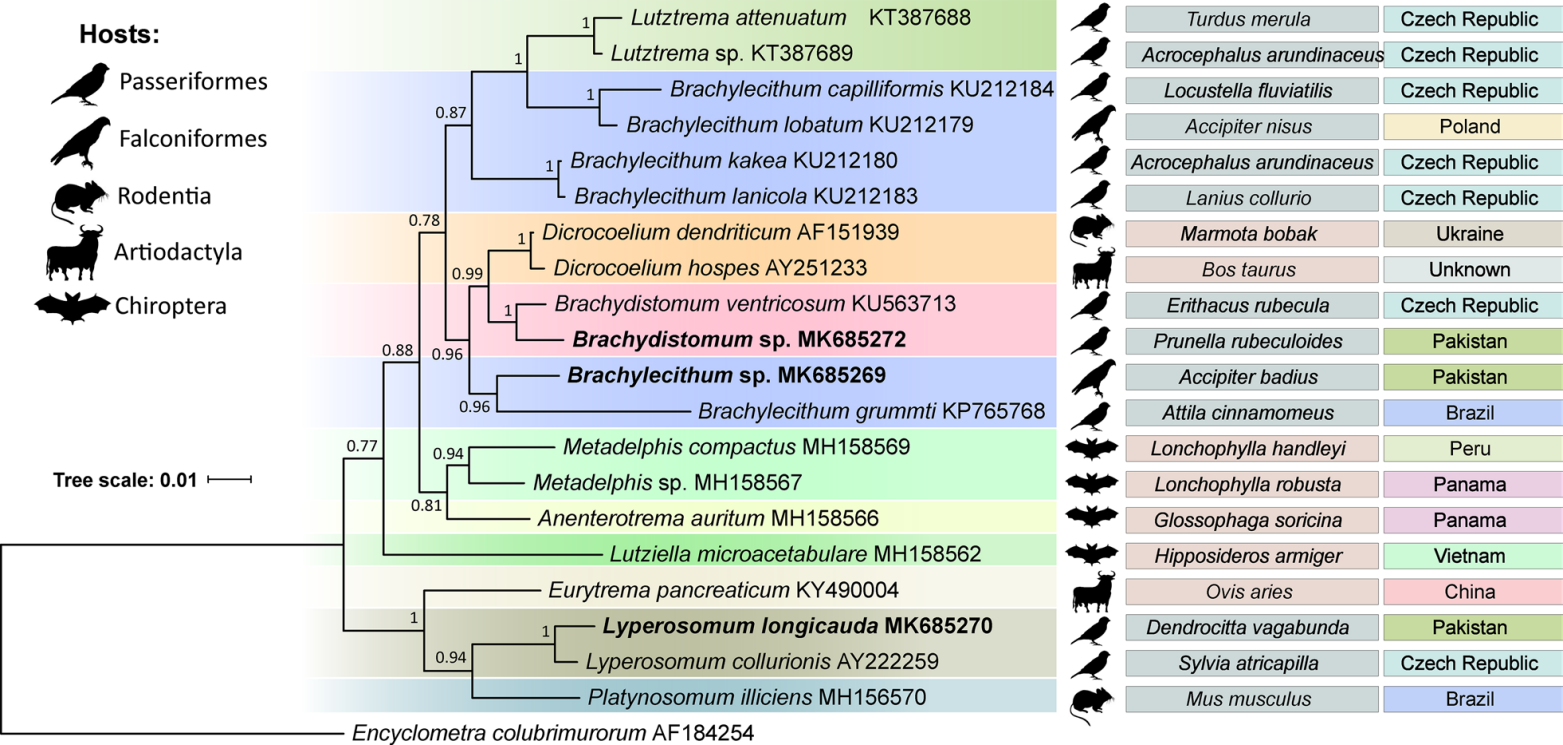
Phylogenetic interrelationships among 20 dicrocoeliids resulting from Bayesian inference (BI) analysis using partial 28S rDNA sequences. Node labels indicate posterior probabilities (> 70%). Host groups, name and geographical regions are shown to the right from each digenean taxon. The newly generated sequences are indicated in bold. *Encyclometra colubrimurorum* was used as the outgroup

The sequenced region of the 28S gene was 1323 bp long for *L. longicauda*, 1274 bp for *Brachylecithum* sp. and 1371 bp for *Brachydistomum* sp. Sequences of two isolates of *L. longicauda* were identical. Bayesian analysis based on partial 28S rDNA placed our specimens close to other members of the three corresponding genera, i.e. *Lyperosomum* Looss, 1899; *Brachydistomum* Travassos, 1944 and *Brachylecithum* Shtrom, 1940 thus providing additional confirmation of their identifications. The analysis placed *L. longicauda* (the type-species) close to *Lyperosomum collurionis* (Skrjabin & Isaichikov, 1927) with maximum nodal support (bpp = 1) in a clade also containing *Platynosomum illiciens* (Braun, 1901) and *E. pancreaticum* (Janson, 1889) (Fig. 1). Our specimens identified as *Brachydistomum* sp. grouped together with *Brachydistomum ventricosum* (Rudolphi, 1802) (bpp = 1) in a clade that also contained species of *Dicrocoelium* Dujardin, 1845. The clade containing *Brachylecithum grummti* Odening, 1964 and *Brachylecithum* sp. (present study) appeared close (bpp = 0.96) to the clade containing species of *Dicrocoelium* and *Brachydistomum* and was not closely related to the remaining members of *Brachylecithum* in the tree, namely the clades *B. lobatum* Railliet, 1900 + *B. capilliformis* Oshmarin, 1952 and *B. kakea* Bhalerao, 1926 + *B. laniicola* Layman, 1926. Recent studies documented the paraphyletic nature of the genus *Brachylecithum* [1, 15]. Tkach et al. [1] suggested that *B. grummti* likely represents a separate genus, but underlined that formally establishing this new genus requires a thorough additional morphological analysis. Our study added an additional species to the *B. grummti* clade thus potentially strengthening the case for establishing a new genus and providing additional molecular and morphological evidence.

### Gene organization, size and nucleotide content of mitogenomes

**Fig. 2:**
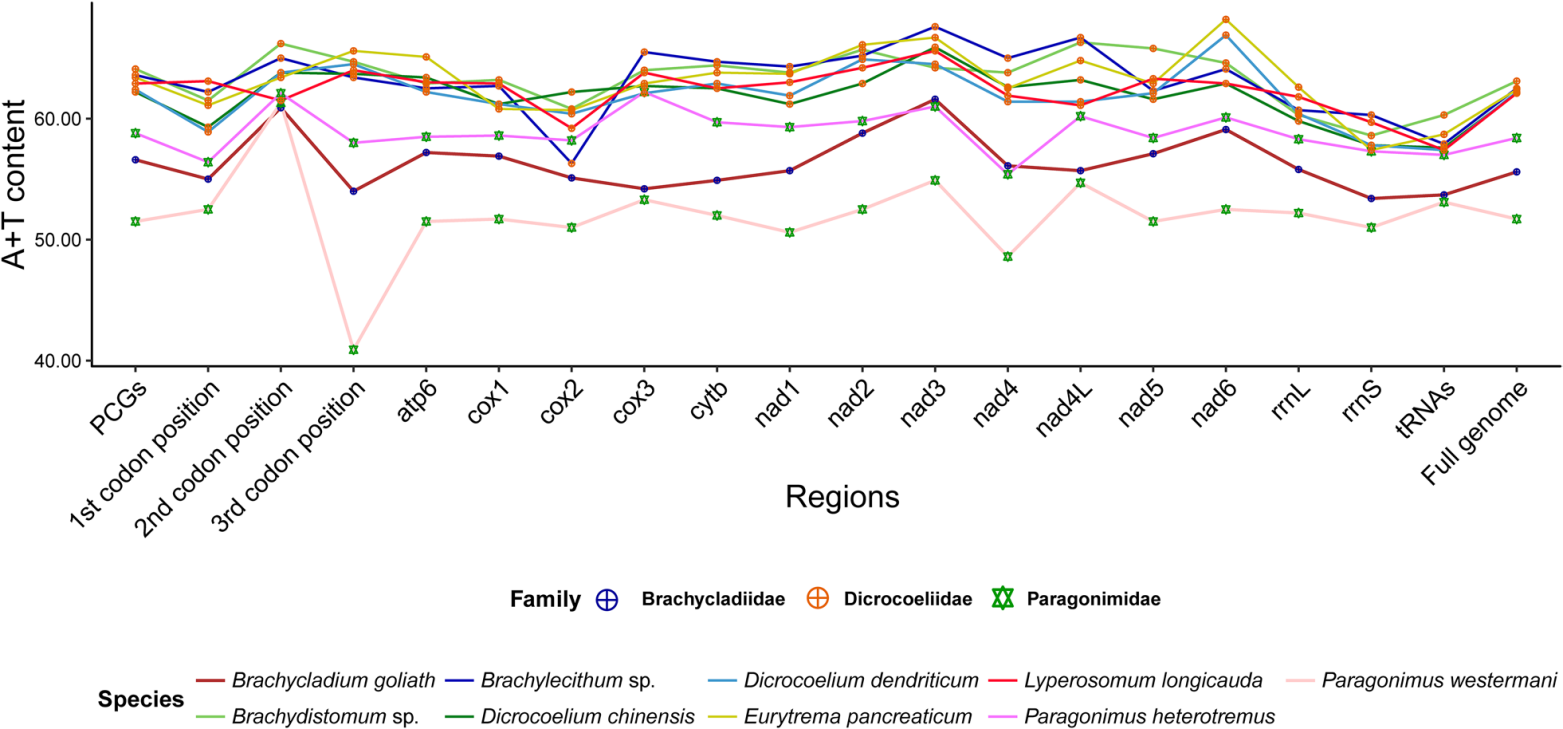
A + T content (%) of genes, each codon position of PCGs and complete or nearly complete mitochondrial genomes of the three newly sequenced samples in this study and other xiphidiatans downloaded from GenBank. Line color and symbols indicate xiphidiatan species and families, respectively

The complete circular mt genome of *L. longicauda* was 14,567 bp (GenBank: MK685274) while the nearly complete mitogenome of *Brachydistomum* sp. was 13,353 bp (GenBank: MK685273) and of *Brachylecithum* sp. was 13,275 bp (GenBank: MK685271) in size. We failed to obtain high quality sequences of the non-coding regions and two tRNAs (*trnE* and *trnG*) of the mt genomes of *Brachylecithum* sp. and *Brachydistomum* sp., most likely due to the presence of multiple regions of repetitive motifs which terminated sequencing. Similar regions in other studies of mt genomes have also been shown as problematic [46, 47]. Therefore, the PacBio single-molecule real-time sequencing method has been used by researchers in recent studies to characterize long and difficult to sequence repetitive regions of flatworm mitogenomes [48, 49]. Nonetheless, the coding regions of the mt genomes of *Brachylecithum* sp. and *Brachydistomum* sp. sequenced in the present study were sufficient for the characterization of their mt genomes and phylogenetic implications. The gene order of all three newly sequenced dicrocoeliid mt genomes was identical to that of previously published dicrocoeliids and several other digeneans including xiphidiatans (*Paragonimus* spp.). The nucleotide composition in dicrocoeliid mt genomes showed a significant bias toward T. The overall A + T content in the mt genomes of *L. longicauda*, *Brachydistomum* sp. and *Brachylecithum* sp. was 62.1%, 63.1% and 62.6%, respectively. This is very close to the proportions seen in *D. chinensis* (62.11%), *D. dendriticum* (62.18%) and *E. pancreaticum* (62.50%). Interestingly, all six dicrocoeliid mt genomes have a higher A + T content in their individual genes, each codon position (1st, 2nd and 3rd) and the entire mt genome than other xiphidiatans with published mitogenomes, e.g. *Brachycladium goliath* (55.6%) (Brachycladiidae), *Paragonimus heterotremus* (58.4%) and *Paragonimus westermani* (51.7%) (Paragonimidae) (Fig. 2).

### Protein-coding genes and codon usage

Concatenated 12 PCGs were 10,126 bp, 10,154 bp and 10,160 bp in size, with A + T contents being 62.9%, 64.1% and 63.6% in mt genomes of *L. longicauda*, *Brachydistomum* sp. and *Brachylecithum* sp., respectively (Additional file 3: Table S2). ATG and GTG were the most common start codons for 12 PCGs of three studied avian dicrocoeliid mt genomes with exception of the *nad*2 and *cox*1 of the *Brachylecithum* sp. which used TTG as the start codon. The start codon TTG for the PCGs was observed in the mitogenomes of many other flatworms, for example the *nad*1, *cytb*, *nad*2 and *cox*1 of *Sindiplozoon* sp. [50], the *nad*5 of *Fasciola* sp. [51] and *cytb* and *nad*5 of *D. chinensis* and *D. dendriticum* [19]. Most of the 12 PCGs used the standard TAG or TAA or the abbreviated T as translation stop codons (Table 1). Codon usage and RSCU of the six available dicrocoeliid mitogenomes showed a high proportion of amino acids encoded by guanine and thymine-rich codons (such as Val, Phe, Leu2 and Gly) compared to those encoded by adenosine and cytosine-rich codons (such as Gln, Lys and His). However, there were no significant differences in the proportion of amino acids used for the construction of 12 PCGs across six available dicrocoeliid mitogenomes (Additional file 4: Figure S2).

**Table 1:** Comparison of the annotated mitochondrial genomes of *Brachydistomum* sp., *Brachylecithum* sp. and *Lyperosomum longicauda*

Gene/region	Position (5′-3′)		Length (bp)	Start codon	Stop codon	Anti-codon	Identity (%)
*Brachydistomum* sp./*Brachylecithum* sp./*Lyperosomum longicauda*							Bd-Bl/Bd-Ll/Bl-Ll/A
*cox*3	1/1/1	651/651/645	651/651/645	ATG/ATG/ATG	TAA/TAG/TAA		78.34/62.98/64.82/68.71
*trnH*	691/661/663	764/723/729	74/63/67			GTG/GTG/GTG	55.41/51.35/75.36/60.71
*cytb*	768/724/733	1886/1833/1848	1119/1110/1116	ATG/GTG/ATG	TAG/TAG/TAG		79.54/77.39/76.97/77.97
*nad*4L	1891/1826/1849	2160/2089/2118	270/264/270	ATG/ATG/GTG	TAA/TAA/TAA		72.59/62.64/67.40/67.54
*nad*4	2121/2050/2082	3389/3318/3365	1269/1269/1284	ATG/ATG/GTG	TAG/TAG/TAG		69.74/62.55/66.05/66.11
*trnQ*	3397/3329/3375	3458/3391/3436	62/63/62			TTG/TTG/TTG	84.13/76.19/77.78/79.37
*trnF*	3462/3394/3440	3523/3456/3504	62/63/65			GAA/GAA/GAA	95.24/81.82/81.82/86.29
*trnM*	3524/3457/3503	3584/3517/3565	61/61/63			CAT/CAT/CAT	96.72/74.60/74.60/81.98
*atp*6	3585/3518/3566	4094/4027/4084	510/510/519	ATG/ATG/ATG	TAA/TAG/TAG		58.91/57.36/54.78/57.02
*nad*2	4101/4028/4091	4967/4900/4963	867/873/873	ATG/TTG/ATG	TAG/TAG/TAG		65.29/60.54/58.59/61.48
*trnV*	4985/4913/4974	5048/4976/5043	64/64/70			TAC/TAC/TAC	75.38/81.43/74.29/77.03
*trnA*	5055/4980/5050	5122/5041/5115	68/62/66			TGC/TGC/TGC	73.53/73.53/74.24/73.77
*trnD*	5132/5045/5116	5200/5111/5184	69/67/69			GTC/GTC/GTC	77.14/68.06/71.01/72.07
*nad*1	5202/5113/5185	6108/6016/6090	907/904/906	GTG/GTG/ATG	T/T/TAA		75.52/71.40/70.63/72.52
*trnN*	6109/6020/6103	6174/6083/6171	66/64/69			GTT/GTT/GTT	69.12/75.36/69.57/71.35
*trnP*	6184/6098/6177	6254/6163/6249	71/66/73			TGG/TGG/TGG	64.79/63.64/67.12/65.18
*trnI*	6270/6164/6256	6334/6227/6321	65/64/66			GAT/GAT/GAT	80.00/83.33/74.24/79.19
*trnK*	6344/6244/6333	6410/6310/6399	67/67/67			CTT/TTT/CTT	65.71/67.14/65.22/66.02
*nad*3	6411/6310/6402	6759/6658/6747	349/349/346	ATG/GTG/GTG	T/T/T		60.74/57.31/65.90/61.32
*trnS*1	6760/6659/6748	6818/6718/6804	59/60/57			TCT/GCT/GCT	65.00/66.10/75.00/68.70
*trnW*	6850/6721/6806	6914/6780/6870	65/60/65			TCA/TCA/TCA	70.77/81.54/69.23/73.85
*cox*1	6923/6784/6891	8479/8334/8438	1557/1551/1548	ATG/TTG/ATG	TAG/TAG/TAG		79.77/75.21/79.13/78.04
*trnT*	8502/8376/8451	8564/8437/8518	63/62/68			TGT/TGT/TGT	70.77/69.12/66.67/68.85
*rrnL*	8567/8439/8519	9546/9414/9508	980/976/990				77.36/73.69/74.00/75.02
*trnC*	9547/9415/9509	9612/9477/9570	66/63/62			GCA/GCA/GCA	80.30/74.24/73.02/75.85
*rrnS*	9613/9478/9571	10324/10226/10304	712/749/734				74.74/68.18/72.35/71.76
*cox*2	10325/10227/10305	10957/10865/10895	633/639/591	ATG/ATG/ATG	TAG/TAG/TAG		56.99/62.11/58.07/59.06
*nad*6	10964/10879/ 10916	11413/11340/11365	450/462/450	GTG/ATG/ATG	TAG/TAG/TAG		59.05/64.44/65.52/63.00
*trnY*	11416/11346/11366	11488/11407/11428	73/62/63			GTA/GTA/GTA	58.90/65.75/61.54/62.07
*trnL*1	11497/11408/11433	11560/11474/11495	64/67/63			TAG/TAG/TAG	52.17/69.23/69.12/63.51
*trnS*2	11566/11480/11497	11634/11543/11562	69/64/66			TGA/TGA/TGA	47.83/62.86/55.22/55.30
*trnL*2	11639/11549/11564	11702/11612/11629	64/64/66			TAA/TAA/TAA	73.85/74.24/76.12/74.74
*trnR*	11711/11629/11634	11778/11695/11694	68/67/61			TCG/TCG/TCG	54.93/57.35/52.24/54.84
*nad*5	11782/11698/11697	13353/13275/13274	1572/1578/1578	GTG/ATG/ATG	TAA/TAG/TAA		64.50/60.23/60.45/61.73
*trnG*	−/−/13277	−/−/13340	−/−/64			−/−/TCC	
SNCR	−/−/13341	−/−/13738	−/−/398				
*trnE*	−/−/13739	−/−/13800	−/−/62			−/−/TTC	
LNCR	−/−/13801	−/−/14567	−/−/767		TAA/TAG/TAA		

*Abbreviations*: Bd, *Brachydistomum* sp.; Bl, *Brachylecithum* sp.; Ll, *Lyperosomum longicauda*; A, average identity values (%) of the three dicrocoeliids

### Transfer and ribosomal RNA genes

Twenty-two transfer RNA (tRNA) genes were found in the complete mt genome of *L. longicauda* and 20 (excluding *trnG* and *trnE*) were found in the nearly complete mt genomes of *Brachydistomum* sp. and *Brachylecithum* sp. Their total concatenated length was 1434 bp, 1320 bp and 1273 bp for *L. longicauda*, *Brachydistomum* sp. and *Brachylecithum* sp., respectively. All tRNA genes had the standard anticodons observed in other flatworms, with the exception of *trnS*1 in *Brachydistomum* sp. and *trnK* in *Brachylecithum* sp. where anticodon TCT was found for *trnS*1 and TTT for *trnK*. This modification of anticodons, supposedly a homoplasy [50], from GCT to TCT (in *trnS*1) and CTT to TTT (in *trnK*) was also found in some other flatworms, e.g. *Hypoderaeum conoideum* (Trematoda: Echinostomatidae) [52] and *Khawia sinensis* (Cestoda: Caryophyllidea) [53]. The secondary structures of tRNAs were similar to those in the corresponding genes in *E. pancreaticum* [20]; all tRNAs except for *trnS*1 (AGN) and *trnS*2 (UCN), could be folded into the typical cloverleaf secondary structure. Similar to other dicrocoeliids, the two ribosomal RNA genes (*rrnL* and *rrnS*) in the species examined in our study, were located between *trnT* and *cox*2 for each species. The A + T content in the *rrnL* gene (60.3–61.8%) was higher than in the *rrnS* gene (58.6–60.3%) across all three species.

### Non-coding regions

The complete mt genome of *L. longicauda* contained two non-coding regions (NCRs); a short non-coding region (SNCR) (398 bp) located between *trnG* and *trnE* and a long non-coding region (LNCR) (769 bp) located between *trnE* and *cox*3. Both NCRs were located in the same position as in the previously published mitogenomes of mammalian dicrocoeliids. The LNCR of *L. longicauda* mt genome contained three sets of identical tandem repeats (TRs); two 80 bp long tandem repeats and one 36 bp long tandem repeat. The SNCR lacks any tandem repeat sequences, but is capable of forming a stem-looped structure like that of tandem repeats of LNCR, as predicted by Mfold (Additional file 5: Figure S3). The A + T content in SNCR (68.3%) of *L. longicauda* mt genome is higher than that in LNCR (62.8%).

### Sliding window analysis and nucleotide diversity among dicrocoeliid mitogenomes

**Fig. 3:**
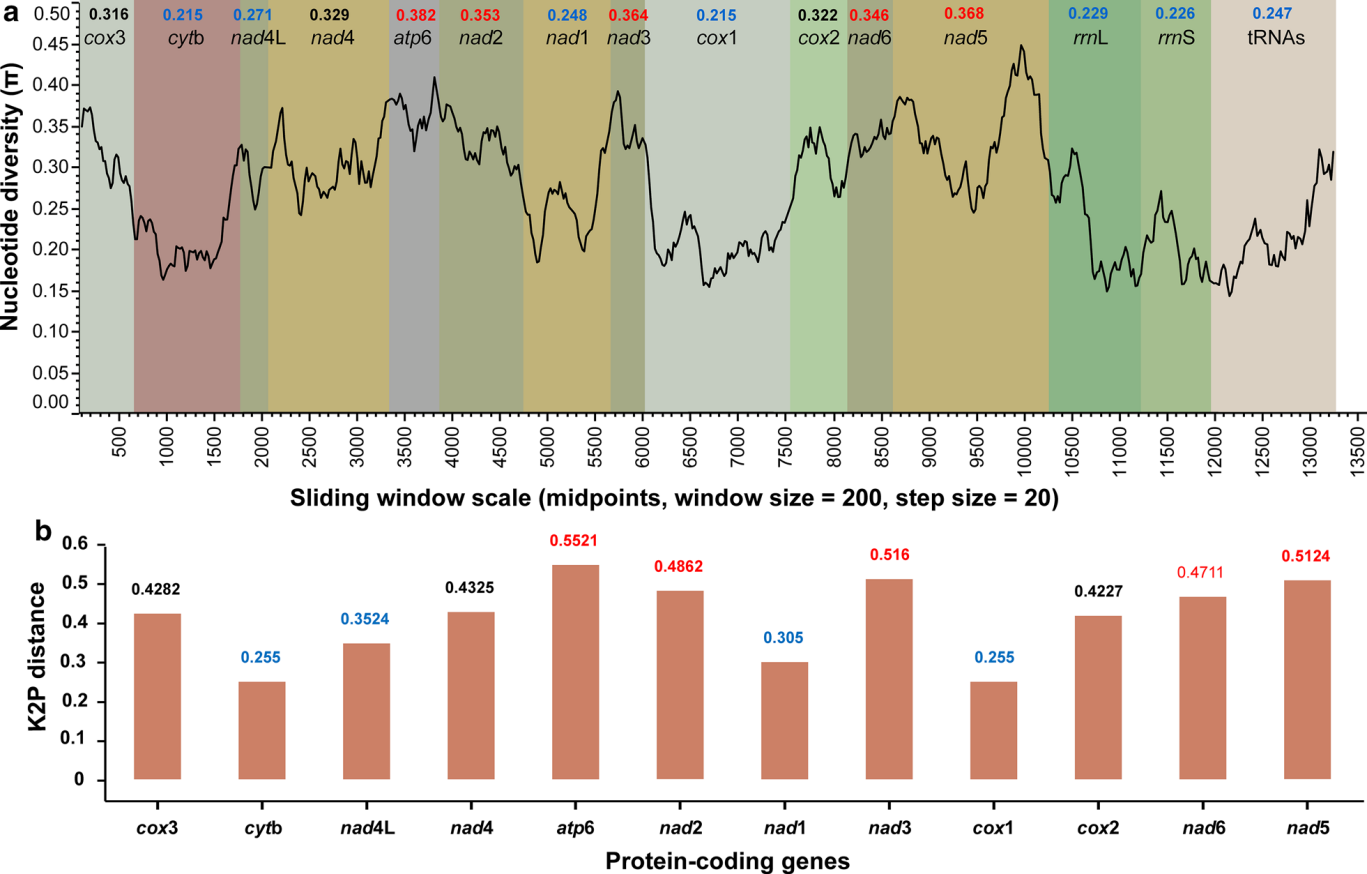
Genetic analyses among six dicrocoeliid mitogenomes. **a** Sliding window analysis of the alignment of 12 protein-coding genes (PCGs), 2 rRNAs and 18 coalescent tRNAs (*trnE* and *trnG* were removed as we did not obtained their sequences for our two samples). The black line represents nucleotide variation in a window of 200 bp (step size = 20 bp, with the value inserted at its mid-point). Gene boundaries are indicated by color with mean variation ratio per gene shown above each gene. **b** The Kimura-2-parameter distance (K2P) among 12 PCGs of dicrocoeliid mitogenomes. The highest and lowest Pi values and K2P distance are indicated by red and blue color, respectively

The sliding window analysis was conducted using an alignment of concatenated mt PCGs, rRNAs and tRNAs (except *trnG* and *trnE*) of all six dicrocoeliid mitogenomes to determine the most conserved and most variable genes. The plot revealed a high nucleotide diversity among six mitogenome sequences with Pi values ranging from 0.144 to 0.449 (window size = 200 bp, step size = 20). Genes with relatively high sequence variability included *atp*6 (0.382), *nad*5 (0.368), *nad*3 (0.364) and *nad*2 (0.353), while *cox*1 and *cytb* (0.215), *rrnS* (0.226), *rrnL* (0.229), tRNAs (0.247) and *nad*1 (0.248) showed relatively lower sequence variability (Fig. 3a). Similarly, *atp*6 had the highest Kimura-2-parameter (K2P) genetic distance across the 12 PCGs among the six dicrocoeliid mitogenomes, followed by *nad*5 and *nad*3 (Fig. 3b).

**Fig. 4:**
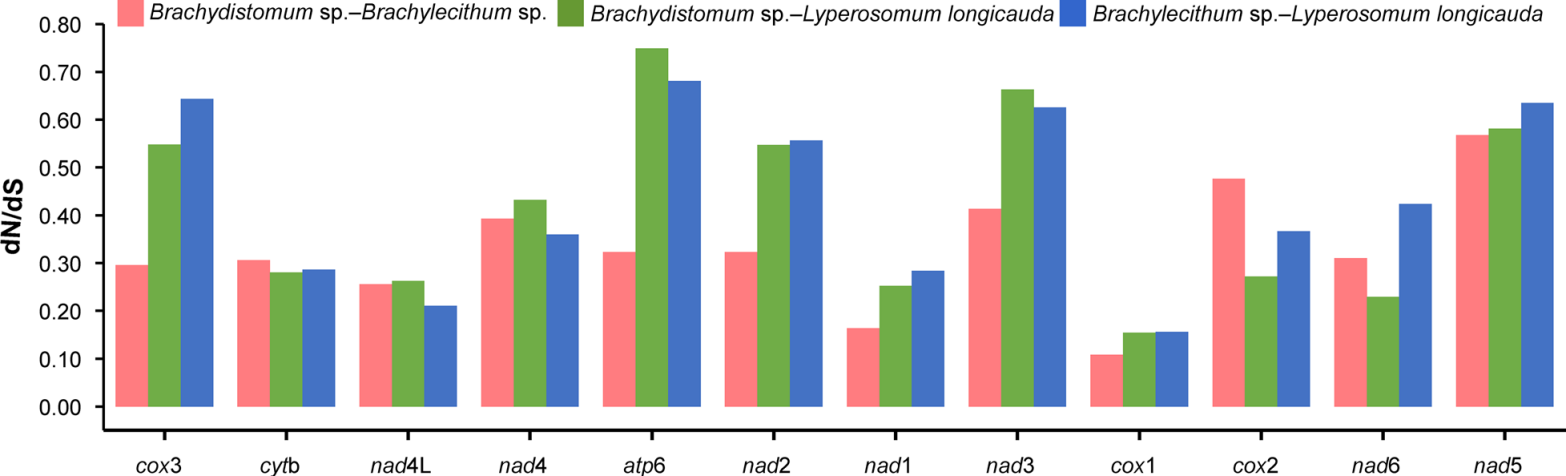
Ratios of non-synonymous to synonymous (dN/dS) substitution rates calculated from individual protein-coding genes of three newly sequenced *Brachydistomum* sp., *Brachylecithum* sp. and *Lyperosomum longicauda* mitogenomes

This was further corroborated by the average sequence identity (Table 1) and dN/dS ratio analysis among the 12 PCGs of the three newly obtained dicrocoeliid mitogenomes. These analyses produced similar results where the average sequence identity was the highest across *cox*1 (78.04%), *cytb* (77.97%) and *nad*1 (72.52%) and the lowest across *atp*6 (57.02%), *cox*2 (59.06%), *nad*3 (61.32%), *nad*2 (61.48%) and *nad*5 (61.73%). Likewise, the highest non-synonymous substitution rate (dN) was observed in *atp*6, while *cox*1 had the lowest dN value of the 12 PCGs (Fig. 4).

Thus, our analyses showed the comparatively fast mutation rate of *atp*6, *nad*5, *nad*3 and *nad*2 while *cox*1, *cytb* and *nad*1 are evolving comparatively slowly in the Dicrocoeliidae. Although *cox*1, *cytb*, *nad*1 and two mt rRNAs are often used as barcodes and have been extensively used for species identification and population genetics in trematodes, we suggest that fast evolving *atp*6, *nad*5, *nad*3 and *nad*2 would be better markers for analyzing relationships among dicrocoeliids (and likely, other digeneans) at lower taxonomic levels. Similar fast-evolving mt genes have been proposed as better molecular markers for analyzing relationships among closely related species of other flatworms in recent studies [50, 54].

### Phylogeny of the Dicrocoeliidae based on mt genome sequences

**Fig. 5:**
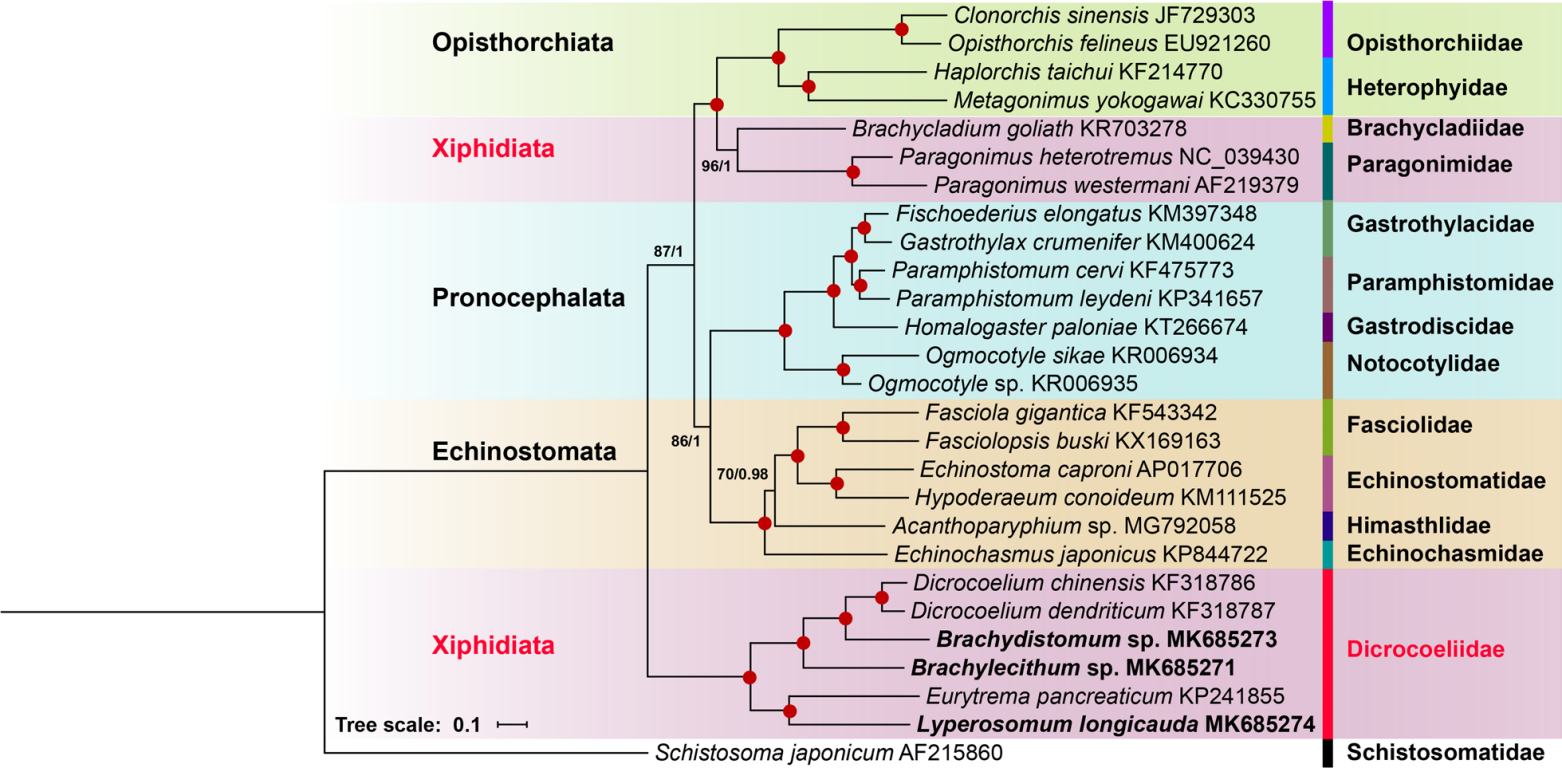
Phylogeny of the order Plagiorchiida based on available mitochondrial genome data. The phylogram was constructed based on Bayesian inference (BI) and maximum likelihood (ML) methods using concatenated nucleotide sequences of protein-coding genes, rRNAs and tRNAs of 27 digenean mitogenomes. Statistical support values (Bootstrap/posterior probability) of ML/BI analysis are shown above the nodes. Circles indicate ML/BI = 100/1.0; other values are given above the nodes. Suborders and families are highlighted by individual colors. *Schistosoma japonicum* (Diplostomida: Schistosomatidae) was used as the outgroup

Both ML and BI analyses produced phylogenetic trees with the same branch topologies and only minor differences in statistical support values for some nodes (Fig. 5). Similarly, the phylogenetic tree based on concatenated amino acid sequences (BI analysis) of 11 PCGs was consistent with that resulted from nucleotide sequences (Additional file 6: Figure S4). The only notable difference was the position of the clade containing members of the Gastrothylacidae, Paramphistomidae, Gastrodiscidae and Notocotylidae which clustered together in a separate clade close to the clade containing members of the Opisthorchiidae, Heterophyidae, Brachycladiidae and Paragonimidae (Additional file 6: Figure S4). The position of the Dicrocoeliidae clade as well as the internal branch topology within the Dicrocoeliidae were identical in the phylogenies based on the nucleotide and amino acid sequence alignments. The general tree topology and positions of included families were consistent with previous studies based on mitogenome sequences [19, 20, 55]. The phylogenetic tree supported the monophyly of all families represented in the analysis by more than a single mt genome. Within the Dicrocoeliidae, all six dicrocoeliids belonging to five genera clustered together with maximum nodal support (bootstrap values = 100 and bpp = 1). The position of *Brachydistomum* sp. and *Brachylecithum* sp. was consistent with their respective placement in the present as well as in previous phylogenetic analyses based on 28S rDNA sequences [1, 15]. The tree also showed that the family Paragonimidae (represented by *Paragonimus* spp.) was genetically closer to the Brachycladiidae than to the Dicrocoeliidae while some of the previously published data, based on nuclear ribosomal genes, documented the close genetic relationship among the Paragonimidae and the Dicrocoeliidae, where both families were classified within the superfamily Gorgoderoidea [12].

Although discordance between phylogenies based on shorter nuclear sequences and mitogenomes is not uncommon [18], some previous phylogenetic analyses based on 28S rDNA [16, 56] also did not support close relationships between the Paragonimidae and other families classified within the Gorgoderoidea *sensu* Curran, Tkach & Overstreet, 2006. Moreover, the position of the Dicrocoeliidae in the mitogenome-based tree is also problematic since it is positioned outside the clade uniting other xiphidiatan trematodes (Brachycladiidae and Paragonimidae), thus suggesting that the content of the Xiphidiata as defined by Olson et al. [12] and the interrelationships between its constituent families may need to be reconsidered with more sequence data, e.g. ultra-conserved elements (UCEs) [18].

## Conclusions

The present study reports results of the analysis of complete, or nearly complete, mt genomes of three avian dicrocoeliids representing three genera *Brachylecithum*, *Brachydistomum*, and *Lyperosomum*. We analyzed phylogenetic affinities of these taxa within the family Dicrocoeliidae using partial sequences of the nuclear large ribosomal subunit (28S) RNA gene and a nearly complete set of mt genes. The phylogeny based on the 28S gene provided additional evidence for the paraphyletic nature of *Brachylecithum* and revealed an additional sister species to *B. grummti* which likely represents a separate genus. Our analysis of the combined nucleotide diversity, Kimura-2-parameter distances, non-synonymous/synonymous substitutions ratios and average sequence identity among dicrocoeliid mitogenomes suggest that *atp*6, *nad*5, *nad*3 and *nad*2 genes are better molecular markers for differentiation and population level studies than the commonly used *cox*1 and *nad*1 genes for dicrocoeliids. Furthermore, the phylogenetic position of the family Dicrocoeliidae (outside the clade uniting other xiphidiatan trematodes) within the order Plagiorchiida based on all mitochondrial genes (except *trnG* and *trnE*) cautiously suggests that the content of the Xiphidiata may need to be reconsidered with more sequence data. However, considering the high mutation rate and the possible effect of mutation saturation on the results of phylogenetic analyses at higher taxonomic levels, we abstain from suggesting systematic changes at this point.

## Supplementary information

**Additional file 1: Figure S1.** Representatives of the three studied dicrocoeliid species. **a** *Brachydistomum* sp. **b** *Brachylecithum* sp. **c** *Lyperosomum longicauda. Scale-bars*: 1 mm.

**Additional file 2: Table S1.** Sequences of primers used to amplify and sequence the mitochondrial genomes of *Lyperosomum longicauda*, *Brachydistomum* sp., and *Brachylecithum* sp.

**Additional file 3: Table S2.** Nucleotide composition and skewness of three newly sequenced dicrocoeliids mitochondrial genomes.

**Additional file 4: Figure S2.** Relative synonymous codon usage (RSCU) for the 12 protein-coding genes of six dicrocoeliids mitogenomes. Codon families are labeled on the x-axis. Values on the top of the bars indicate percentage of each amino acid used for the construction of 12 protein-coding genes.

**Additional file 5: Figure S3.** Secondary structures of the short non-coding region (SNCR) and tandem repeats (TRs) in the large non-coding regions (LNCR) in the mitogenome of *Lyperosomum longicauda.*

**Additional file 6: Figure S4.** Phylogeny of the order Plagiorchiida based on Bayesian inference (BI) using concatenated amino acid sequences of 12 mitochondrial protein-coding genes. Statistical support values (posterior probability) of BI analysis are shown above the nodes. *Schistosoma japonicum* (Diplostomida: Schistosomatidae) was used as the outgroup.

## Data Availability

The datasets supporting the findings of this article are included within the article and its additional files. The nuclear 28S rDNA nucleotide sequences generated in this study for *L. longicauda*, *Brachydistomum* sp. and *Brachylecithum* sp. were deposited in the GenBank database under the accession numbers MK685270, MK685272 and MK685269, respectively. The mitogenomic sequences of *L. longicauda*, *Brachydistomum* sp. and *Brachylecithum* sp. are available in GenBank under the accession numbers MK685274, MK685273 and MK685271, respectively.

## Abbreviations

BI: Bayesian inference
ML: Maximum likelihood
MCMC: Metropolis-coupled Markov chain Monte Carlo
PCGs: Protein-coding genes
tRNAs: Transfer RNAs
rRNAs: Ribosomal RNAs
RSCU: Relative synonymous codon usage
dN/dS: Non-synonymous/synonymous
TRs: Tandem repeats
NCRs: Non-coding regions
SNCR: Short non-coding region
LNCR: Long non-coding region
K2P: Kimura-2-parameter
