# Supplementary figures and images for: Molecular phylogenetics and mitogenomics of three avian dicrocoeliids (Digenea: Dicrocoeliidae) and comparison with mammalian dicrocoeliids
Source: Parasit Vectors. 2020 Feb 13;13:74. doi: 10.1186/s13071-020-3940-7 (PMC7020495; doi:10.1186/s13071-020-3940-7)

**a**

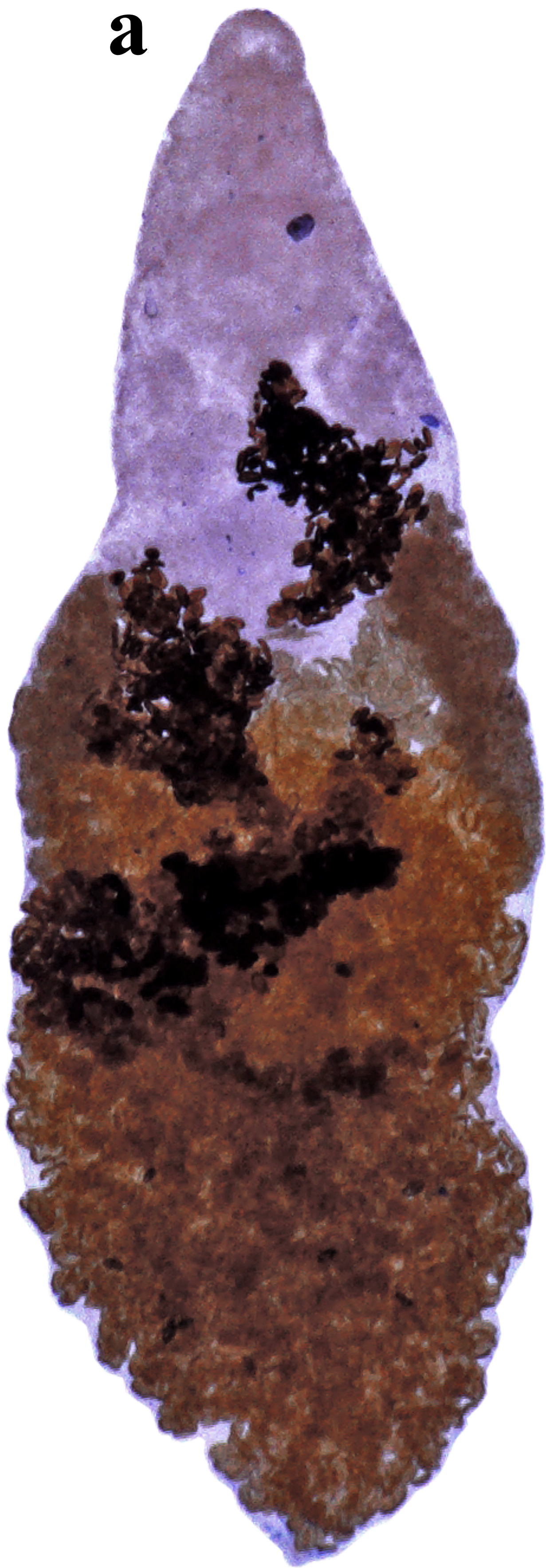

**b**

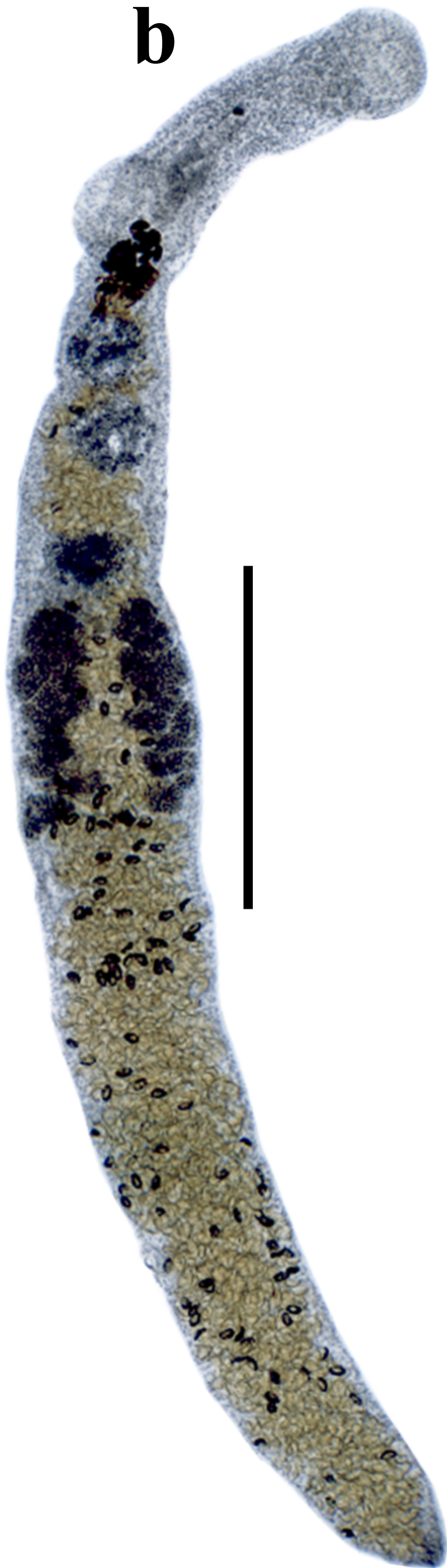

**c**

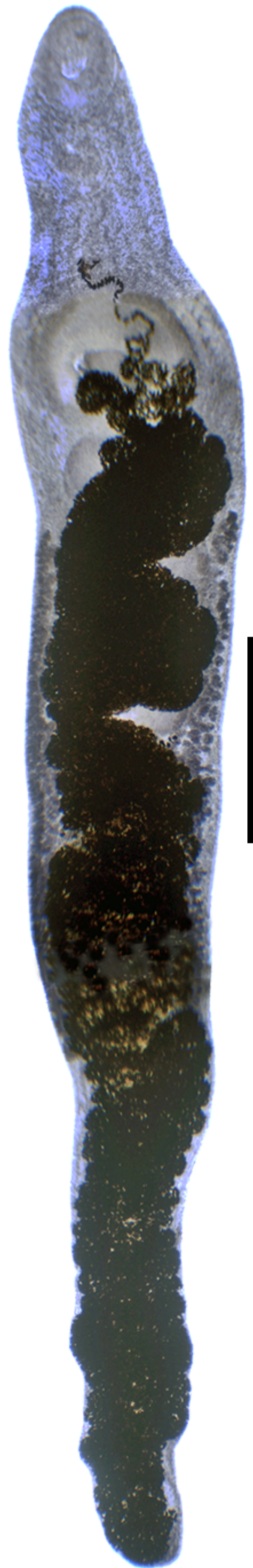

Supplement: Supplementary file 1 — Additional file 1: Figure S1. Representatives of the three studied dicrocoeliid species. a Brachydistomum sp. b Brachylecithum sp. c Lyperosomum longicauda. Scale-bars: 1 mm. [file 13071_2020_3940_MOESM1_ESM.pdf]

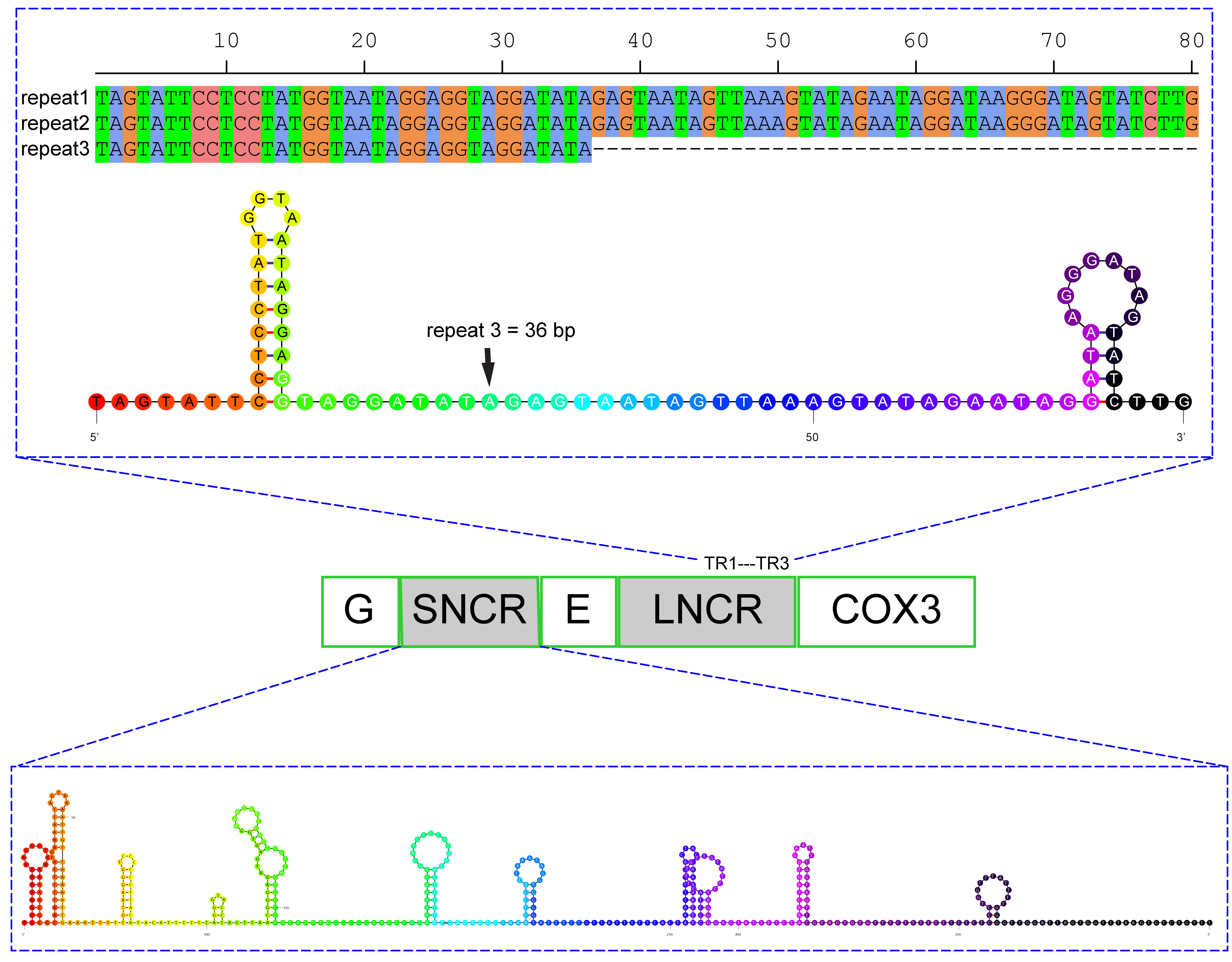

Supplement: Supplementary file 5 — Additional file 5: Figure S3. Secondary structures of the short non-coding region (SNCR) and tandem repeats (TRs) in the large non-coding regions (LNCR) in the mitogenome of Lyperosomum longicauda. [file 13071_2020_3940_MOESM5_ESM.tif]

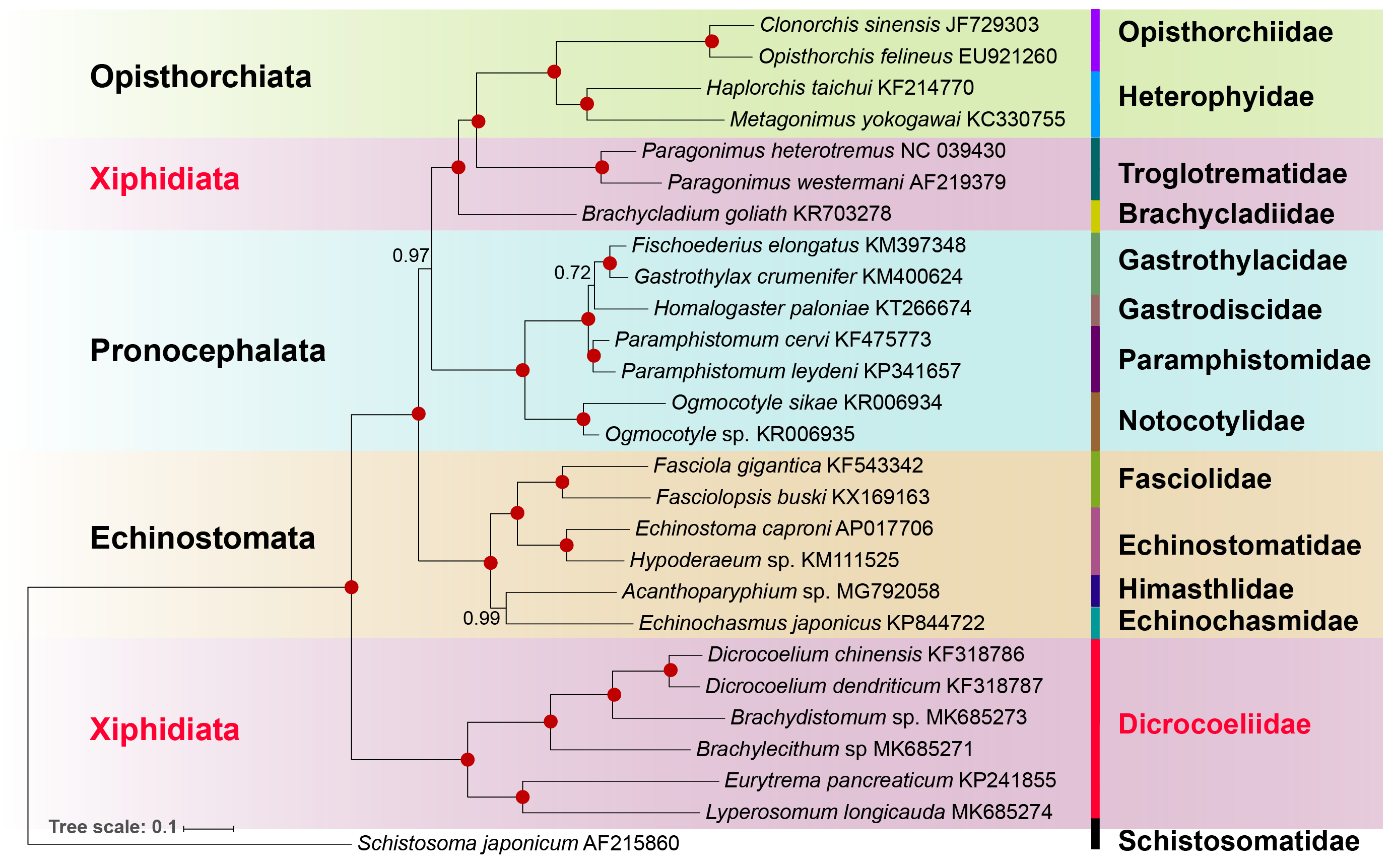

Supplement: Supplementary file 6 — Additional file 6: Figure S4. Phylogeny of the order Plagiorchiida based on Bayesian inference (BI) using concatenated amino acid sequences of 12 mitochondrial protein-coding genes. Statistical support values (posterior probability) of BI analysis are shown above the nodes. Schistosoma japonicum (Diplostomida: Schistosomatidae) was used as the outgroup. [file 13071_2020_3940_MOESM6_ESM.tif]
