# Supplementary material for: Molecular phylogenetics and mitogenomics of three avian dicrocoeliids (Digenea: Dicrocoeliidae) and comparison with mammalian dicrocoeliids
Source: Parasit Vectors. 2020 Feb 13;13:74. doi: 10.1186/s13071-020-3940-7 (PMC7020495; doi:10.1186/s13071-020-3940-7)
Supplement: Supplementary file 2 — Additional file 2: Table S1. Sequences of primers used to amplify and sequence the mitochondrial genomes of Lyperosomum longicauda, Brachydistomum sp., and Brachylecithum sp. [file 13071_2020_3940_MOESM2_ESM.docx]

**Additional file 2: Table S1.** Degenerate primers used for amplification and sequencing of dicrocoeliid mitogenomes.

| **Fragment No.** | **Gene or region** | **Primer name** | **Sequence (5′-3**′**)** | **Annealing**  **temperature** |
| --- | --- | --- | --- | --- |
| F1 | *cyt*b-*nad*4 | DiccytbF | CTKCCTTGGCAYCARATGTCTTAT | 52 |
|  |  | Dicnad4R | GCHARCCADCGCTTVCCNTC |  |
| F2 | *nad*4-*nad*1 | Dicnad4F | GARTCTCCWTATYCKGAGCG | 50 |
|  |  | Dicnad1R | ACCACTAACCARTTCWCTTTC |  |
| F3 | *nad*1-*cox*1 | Dicnad1F | AACTTCGTAAGGGTCCWAAWAAGG | 50 |
|  |  | Diccox1R | CCAAAGACARAACATAATGAAAATGC |  |
| F4 | *cox*1-*rrn*S | Diccox1F | TTTTGGKCATCCTGAGGTTTAT | 57 |
|  |  | DicrrnSR | CTARCRYTACCATGTTACGACTT |  |
| F5 | *rrn*S-*nad*5 | DicrrnSF | CAGTGCCAGCATCCGCGGTTA | 52 |
|  |  | Dicnad4R | TGCTTVSWAAAAAANACHCC |  |
| F6 | *nad*5-*cyt*b | Dicnad5F | ATGCGNGCYCCNACNCCNGTDAG | 52 |
|  |  | DiccytbR | CCGTCGCAGCTCAATAAGACAT |  |

**PCR conditions for degenerate primers:**

Reaction mixture volume: 28 µl

Components:

- 12.5 µl dd H_2_O
- 12.5 µl PrimeStar Max DNA polymerase premix (Takara, Dalian, China)
- 1 µl of each primer
- 1 µl of DNA template

Amplification conditions for long PCR

- initial denaturation at 98°C for 2 min
- 10 cycles of: 92°C for 10 s, 50–57◦C for 30 s, 68 °C for 1 min/kb, followed by
- 92 °C for 2 min
- 22 cycles of: 92 °C for 10 s; 50–57◦C for 30 s; 68 °C for 1 min/kb
- final extension for 10 min at 68 °C
