# Supplementary material for: Molecular phylogenetics and mitogenomics of three avian dicrocoeliids (Digenea: Dicrocoeliidae) and comparison with mammalian dicrocoeliids
Source: Parasit Vectors. 2020 Feb 13;13:74. doi: 10.1186/s13071-020-3940-7 (PMC7020495; doi:10.1186/s13071-020-3940-7)
Supplement: Supplementary file 4 — Additional file 4: Figure S2. Relative synonymous codon usage (RSCU) for the 12 protein-coding genes of six dicrocoeliids mitogenomes. Codon families are labeled on the x-axis. Values on the top of the bars indicate percentage of each amino acid used for the construction of 12 protein-coding genes. [file 13071_2020_3940_MOESM4_ESM.pdf]

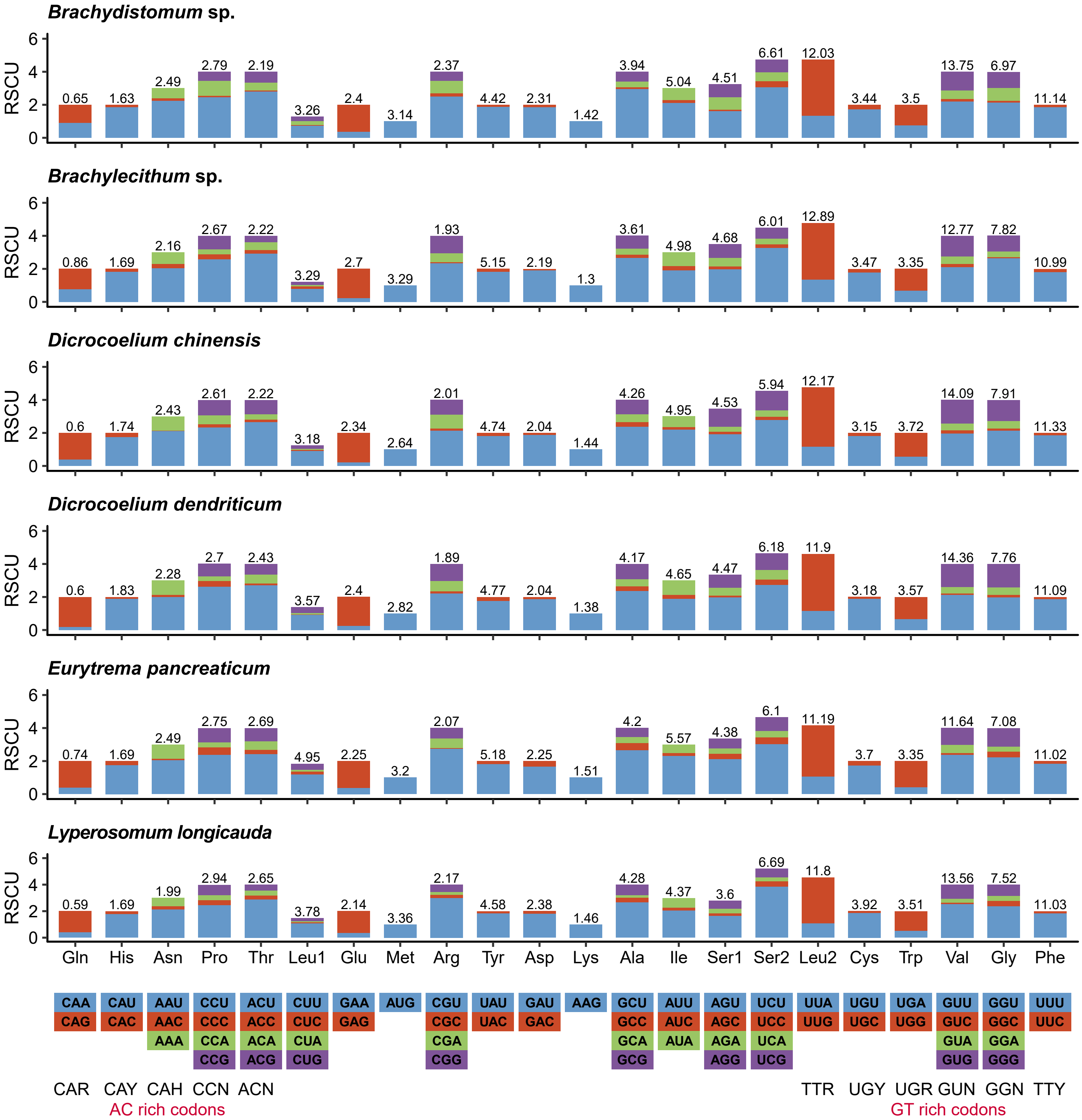

Brachydistomum sp.
Brachylecithum sp.
Dicrocoelium chinensis
Dicrocoelium dendriticum
Eurytrema pancreaticum
Lyperosomum longicauda
RSCU
Gln His Asn Pro Thr Leu1 Glu Met Arg Tyr Asp Lys Ala Ile Ser1 Ser2 Leu2 Cys Trp Val Gly Phe
CAA CAU AAU CCU ACU CUU GAA AUG CGU UAU GAU AAG GCU AUU AGU UCU UUA UGU UGA GUU GGU UUU
CAG CAC AAC CCC ACC CUC GAG CGC UAC GAC GCC AUC AGC UCC UUG UGC UGG GUC GGC UUC
AAA CCA ACA CUA CGA GCA AUA AGA UCA GUA GGA
CCG ACG CUG CGG GCG AGG UCG GUG GGG
CAR CAY CAH CCN ACN
AC rich codons
TTR UGY UGR GUN GGN TTY
GT rich codons
